# Esophageal Cancer Related Gene-4 Is a Choroid Plexus-Derived Injury Response Gene: Evidence for a Biphasic Response in Early and Late Brain Injury

**DOI:** 10.1371/journal.pone.0024609

**Published:** 2011-09-14

**Authors:** Sonia Podvin, Ana-Maria Gonzalez, Miles C. Miller, Xitong Dang, Hannah Botfield, John E. Donahue, Arwa Kurabi, Matthew Boissaud-Cooke, Ryan Rossi, Wendy E. Leadbeater, Conrad E. Johanson, Raul Coimbra, Edward G. Stopa, Brian P. Eliceiri, Andrew Baird

**Affiliations:** 1 Department of Surgery, School of Medicine, University of California San Diego, San Diego, California, United States of America; 2 Department of Neuropharmacology and Neurobiology, School of Clinical and Experimental Medicine, University of Birmingham, Birmingham, United Kingdom; 3 Departments of Neurosurgery and Pathology, Rhode Island Hospital, Warren Alpert Medical School of Brown University, Providence, Rhode Island, United States of America; University of North Dakota, United States of America

## Abstract

By virtue of its ability to regulate the composition of cerebrospinal fluid (CSF), the choroid plexus (CP) is ideally suited to instigate a rapid response to traumatic brain injury (TBI) by producing growth regulatory proteins. For example, Esophageal Cancer Related Gene-4 (Ecrg4) is a tumor suppressor gene that encodes a hormone-like peptide called augurin that is present in large concentrations in CP epithelia (CPe). Because augurin is thought to regulate senescence, neuroprogenitor cell growth and differentiation in the CNS, we evaluated the kinetics of Ecrg4 expression and augurin immunoreactivity in CPe after CNS injury. Adult rats were injured with a penetrating cortical lesion and alterations in augurin immunoreactivity were examined by immunohistochemistry. Ecrg4 gene expression was characterized by *in situ* hybridization. Cell surface augurin was identified histologically by confocal microscopy and biochemically by sub-cellular fractionation. Both Ecrg4 gene expression and augurin protein levels were decreased 24–72 hrs post-injury but restored to uninjured levels by day 7 post-injury. Protein staining in the supraoptic nucleus of the hypothalamus, used as a control brain region, did not show a decrease of auguin immunoreactivity. Ecrg4 gene expression localized to CPe cells, and augurin protein to the CPe ventricular face. Extracellular cell surface tethering of 14 kDa augurin was confirmed by cell surface fractionation of primary human CPe cells in vitro while a 6–8 kDa fragment of augurin was detected in conditioned media, indicating release from the cell surface by proteolytic processing. In rat CSF however, 14 kDa augurin was detected. We hypothesize the initial release and proteolytic processing of augurin participates in the activation phase of injury while sustained Ecrg4 down-regulation is dysinhibitory during the proliferative phase. Accordingly, augurin would play a constitutive inhibitory function in normal CNS while down regulation of Ecrg4 gene expression in injury, like in cancer, dysinhibits proliferation.

## Introduction

Traumatic brain injury (TBI) is often associated with poor clinical outcomes because an over exuberant inflammatory response can cause unintended damage to both injured and non-injured central nervous system (CNS) tissue [1]. In addition, there are biophysical changes in the CNS that restrict functional recoveries. For example, the formation of a glial scar after injury protects neurons from excitotoxic factors [1], [2] but blocks synaptic re-growth and neuroprogenitor cell infiltration from the subventricular zone (SVZ) [3]. Furthermore, edema may disrupt blood-brain (BBB) and blood-cerebrospinal fluid (BCSFB) barriers, compress brain parenchyma [4] and compromise endothelial, choroid plexus epithelial (CPe), ventricular ependymal (Ve) and subarachnoid (SA) cells that normally filter toxins from interstitial fluid, maintain the ionic balance of CSF, and secrete hormones and growth factors to maintain CNS homeostasis [5].

The CPe in particular suffers unique metabolic and structural stresses during the acute stages of CNS injury, which restrict functions that would otherwise restore homeostasis during repair. For example, vasogenic edema after injury increases the volume of the extracellular fluid compartment and causes ventriculomegaly. The resulting mechanical stress [6] and neutrophil invasion [7] increase leakiness of BCSFB tight junctions and sloughing of apical cilia from CPe and Ve cells. Furthermore, disrupted tight junctions impair protein ultrafiltration and lead to increased bulk and osmotic flow of water into CSF [8], [9], [10].

Among the peptides produced in, and regulated by, the CPe [11], [12], augurin is a newly described hormone-like protein that is produced after proteolytic processing of the *esophageal cancer related gene-4* (Ecrg4) holoprotein [13] and is thought to play a role in CNS homeostasis [14], [15]. Previous reports [15] have shown that Ecrg4 gene expression levels in the CNS are highest in CPe and ependyma during both development (www.genepaint.org [16]) and in the adult CNS (www.brain-map.org [17]) and have suggested a possible role for its product augurin in CNS homeostasis. This hypothesis is supported by our observations that knockdown of Ecrg4 gene expression during zebrafish development induced cell over-proliferation in the developing CNS and generated a ventriculomegaly phenotype. In contrast, Ecrg4 overexpression in adult rat brain decreased cell proliferation in the SVZ pool of adult neuroprogenitor cells following a cortical stab model of CNS injury [14].

Here we show that there is a rapid loss of both augurin and Ecrg4 gene expression in CPe after CNS injury. These data support the hypothesis that augurin plays a constitutive inhibitory function in the normal CNS and that its rapid release by and subsequent disappearance from CPe after injury may help trigger and sustain the CNS response to injury by enabling cell proliferation.

## Results

### Augurin is a cell membrane protein

**Figure 1:**
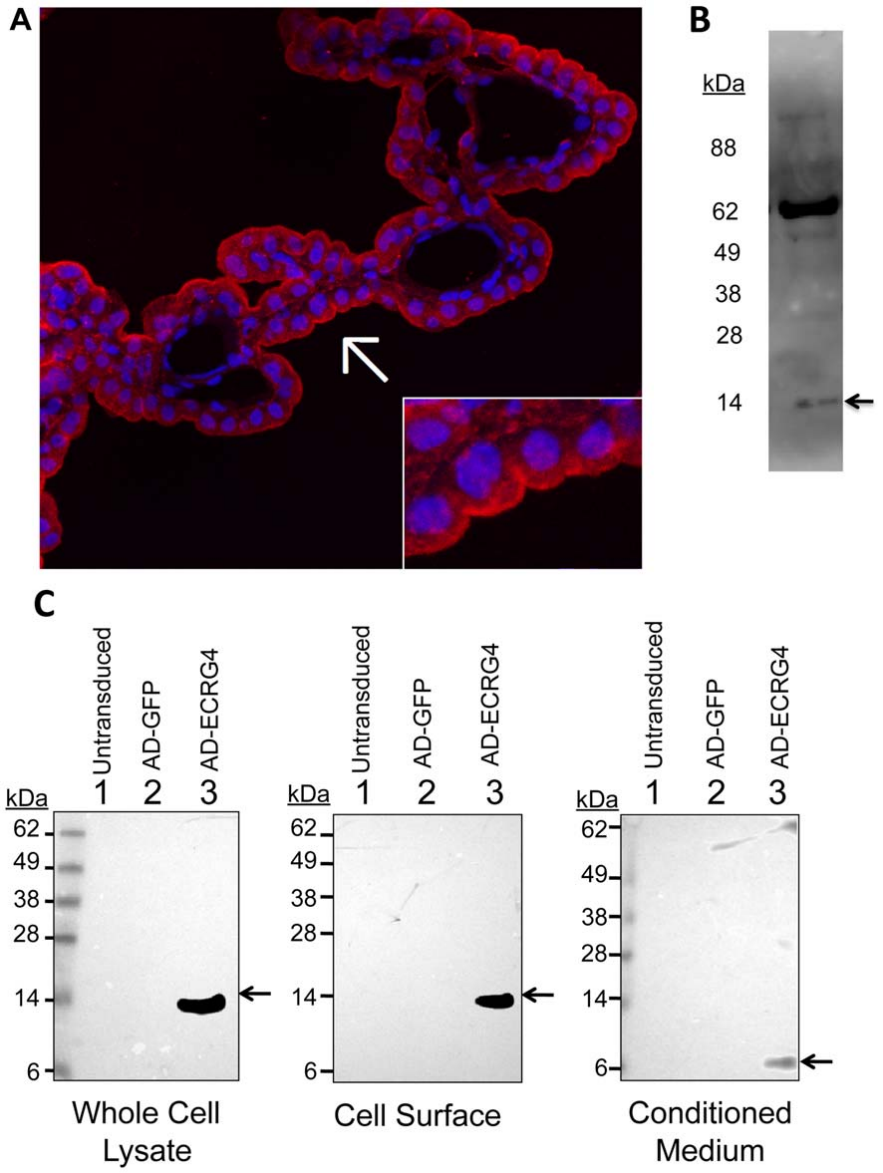
Augurin (ECRG4(31–148)) localizes to the cell surface. Panel A: Immunohistochemical evidence for tethering at the cell surface: The immunoreactive augurin recognized by this antibody in CP extracts was identified as the product of Ecrg4 by immunoblotting and demonstrating the presence of a 14 kDa protein ECRG4(31–148) processed by cleavage of the 30 amino acid leader sequence as we determined previously [13]. Among periventricular cells in the brain, polarized foci of punctated augurin staining were observed at the extracellular ventricular face of the choroid plexus epithelial cell layer (red, arrow) in reference to nuclear counterstain (blue, DAPI). A granular staining pattern is observed in the cytoplasm if the CPe cell layer, which is indicative of regulated vesicular secretion to the cell surface. Panel B: Detection of immunoreactive augurin in rat CSF: Immunoblotting of rat CSF showed a 14 kDa band (arrow) that corresponds to the predicted molecular weight of augurin (ECRG4(31–148)). Panel C: Biochemical evidence for cell surface tethering of augurin: Expression of Ecrg4 in the cultured CPe cells was detected neither by Western blot (left panel) nor by RT-PCR of untransduced cells (not shown). Thus to study Ecrg4 in vitro it was necessary to use an adenovirus vector containing the human Ecrg4 ORF. Protein was extracted from CPe cells 48 hours after no transduction or transduction with AD_GFP_ or AD_ECRG4_. The molecular weight of Ecrg4-derived peptide fragments expressed in whole cell lysates was determined by Western blotting in the left panel. Cell-surface proteins were fractionated by precipitation of Neutravidin-binding of biotinylated proteins on the cell surface (center panel) followed by Western blot analysis. The peptide form secreted from cells was detected by direct Western blotting of conditioned medium (right panel).

Immunohistochemical analyses of augurin in periventricular regions of the brain (Figure 1A, red) showed that, in many instances, the greatest intensity of augurin immunolabeling was polarized (arrow) with significant staining localizing to the ventricular side of CPe cells (red staining compared to DAPI nuclear stain in blue). Furthermore, the pattern of immunoreactivity in the cytoplasm of the CPe was granular, which may be indicative of augurin's presence in secretory vesicles (inset). In contrast augurin immunoreactivity was not detected in CP endothelium or at the abluminal face of CPe. This suggested that, once outside the cell, augurin might be a plasma membrane-associated protein in contact with and with the possibility of secretion into CSF. To determine whether augurin is released into CSF in vivo, we processed rat CSF for immunoblotting and detected a 14 kDa band (Figure 1B) that corresponded to the predicted molecular weight of augurin, the processed 118 amino acid hormone-like peptide encoded by ECRG4(31–148) [18]. A second higher MW band of unknown identity was also detected which may reflect the peptide's preponderance for aggregation (unpublished observation).

To further address the subcellular distribution of augurin in CPe, we biotinylated the cell surface of primary human CPe cells in culture and fractionated the biotinylated proteins via preciptiation with neutravidin beads. We then immunoblotted the cell-surface fraction with an anti-augurin antibody [19]. Cultured CPe cells, like many other cell types in culture that we have examined, do not express endogenous Ecrg4 but could do so following gene delivery with viral or plasmid vectors [14]. After infecting the CPe cells with AD_ECRG4_, a 14 kDa band was detected in whole cell lysates (Figure 1C, left panel, lane 3). Likewise, a 14 kDa protein was detected by immunoblotting of cell-surface proteins indicating that augurin localizes to the cell surface (Figure 1C, middle panel, lane 3). Western blot analysis of the conditioned medium of CPe cells however, in which we previously showed contained augurin (or an immunoreactive fragment of augurin, which was quantified by ELISA [14]), showed an immunoreactive peptide that was approximately 6–8 kDa (Figure 1C, right panel, lane 3). This suggests that, in our in vitro model, peptide cleavage releases (an) augurin fragment(s) from the CPe cell surface. These data establish that in vitro augurin is retained at the cell surface and that cell surface proteolytic processing releases a smaller peptide into media compared to what was detected in rat CSF. This raised the possibility that two differently processed forms of augurin can be shed by CPe cells and could be indicative of two peptide productes each with a distince activity. No immunoreactivity was detected in the whole cell lysates, cell surface fractions or conditioned media of either untransduced or AD_GFP_ transduced cells (Figure 1C, lanes 1 and 2).

### Augurin distribution in choroid plexus changes after CNS injury

**Figure 2:**
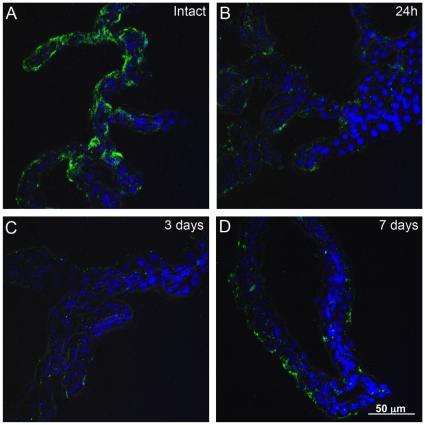
Augurin immunoreactivity decreases after CNS injury. A cortical stab wound decreased augurin protein levels in the contralateral CP as determined by immunohistochemical staining and confocal microscopy. The ophthalmic knife penetrated the rat dorsal cortex to transect the corpus callosum 3 mm deep and lateral to the right lateral ventricle. Immunoreactive augurin protein levels in the contralateral CP were decreased at 24 h (Panel B) and 3 days (Panel C) post injury compared to intact control animals (Panel A). By 7 days after injury augurin protein had returned to approximately the same level as intact controls. Images representative of n = 3 rats.

We used a cortical lesion model of CNS injury [20] to evaluate whether injury effected Ecrg4 gene expression and augurin immunoreactivity in the lateral ventricle CPs. Control and injured rats were killed at 1, 3, and 7 days after performing a 3 mm deep lesion into the right cerebral cortex, lateral to the ventricles. As shown in Figure 2, we observed by confocal microscopy that there was significant diffuse staining in the normal, intact lateral ventricle CP that appeared in many CPe cells to be polarized towards the apical, ventricular facing surface of the epithelial cells and with punctuated areas of immunofluorescent label (Figure 2A). When evaluated 24 hours after injury (Figure 2B) the immunostaining signal was almost absent although there was some immunoreactivity found in isolated foci. Even there however, the overall intensity of staining appeared decreased. By 3 days after the injury (Figure 2C), the immunostaining was still decreased compared to control uninjured animals but it appeared more uniformly distributed throughout the CP as compared to staining 1 day after the lesion. By 7 days, the intensity of staining was now indistinguishable from control brains and there was a combined pattern of polarized apical and focal punctate staining with a more diffuse pattern of augurin immunoreactivity. Taken together, these data suggest that augurin was mobilized immediately after CNS injury and presumably released into CSF.

### Ecrg4 is an injury response gene

**Figure 3:**
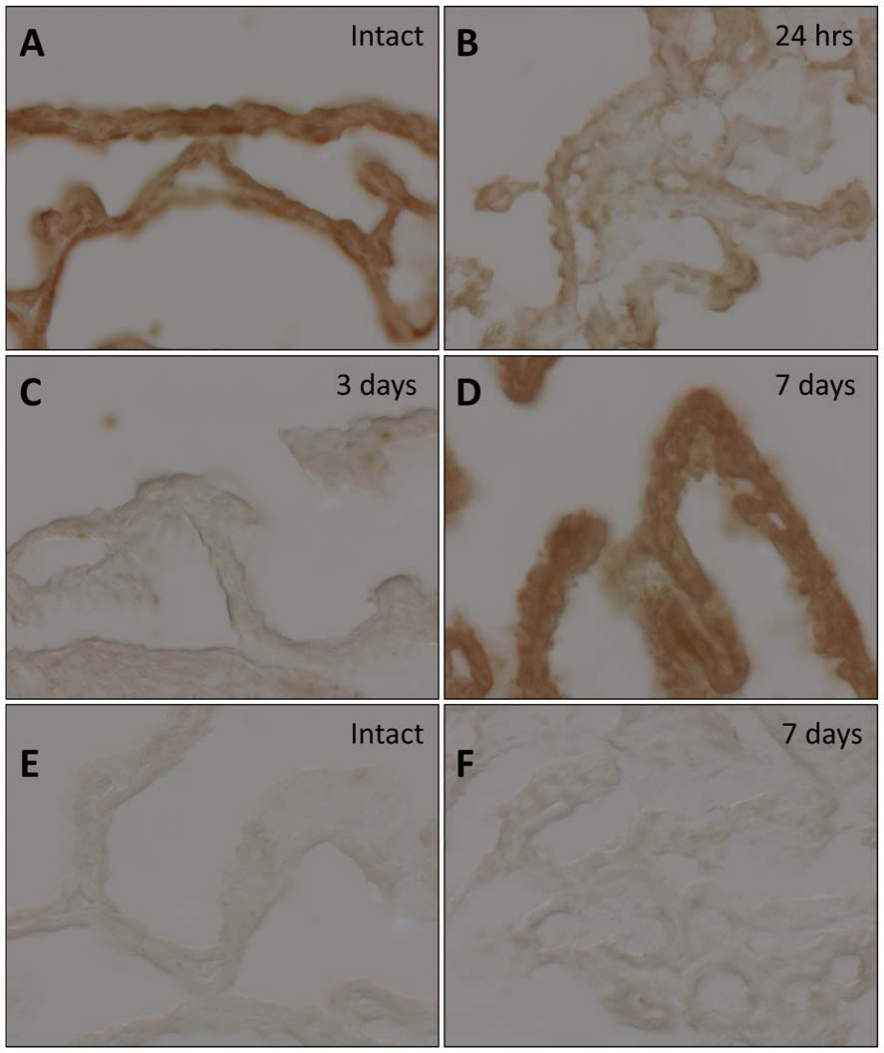
Ecrg4 gene expression decreases after CNS injury. Cortical stab wound decreased gene expression levels in the CP as determined by *in situ* hybridization. The ophthalmic knife penetrated the rat dorsal cortex to transect the corpus callosum 3 mm deep and lateral to the right lateral ventricle and Ecrg4 gene expression evaluated by binding of an Ecrg4 mRNA antisense probe in the contralateral CP in intact rat brains (Panel A), 24 hrs (Panel B) 72 hrs (Panel C) and 7 days (Panel D) post-injury. A synthetic, negative control mRNA probe was generated from the pSPT18-Neo plasmid control backbone and was used to assess background signal in Control (Panel E) and 7 day (Panel F) sections. Images representative of n = 3 rats.

The long term loss of immunoreactive augurin in CPe cells following cortical lesion could be attributed to either (1) continuous and expedited secretion-and-release of augurin depleting intracellular stores or (2) lost augurin through decreased gene expression. Accordingly, we used in situ hybridization to evaluate gene expression in the choroid plexus after injury (Figure 3). An antisense probe to rat Ecrg4 mRNA showed a strong signal in uninjured control rat brains that resembled the pattern observed for immunohistochemical staining (Figure 3A). The signal appeared restricted to CPe cells and as with the protein, the signal was lost 24 hours after injury (Figure 3B) and remained so through to 3 days (Figure 3C) after which, it re-appeared by 7 days (Figure 3D) to levels similar to those in control animals. The signal detected in control animals and 7 days after injury was also deemed specific because no hybridization was detected in CP sections from sham or 7day post lesion animals that were treated with a synthetic control probe (Figure 3E and F). Together, these data suggest that Ecrg4 is an early injury response gene and that the loss of augurin is biphasic: first due to bulk release from the cell surface and intracellular storage vesicles and the second, due to the loss of Ecrg4 gene expression throughout the proliferative phase of injury.

### Augurin distribution in the supraoptic nucleus (SON) does not decrease after CNS injury

**Figure 4:**
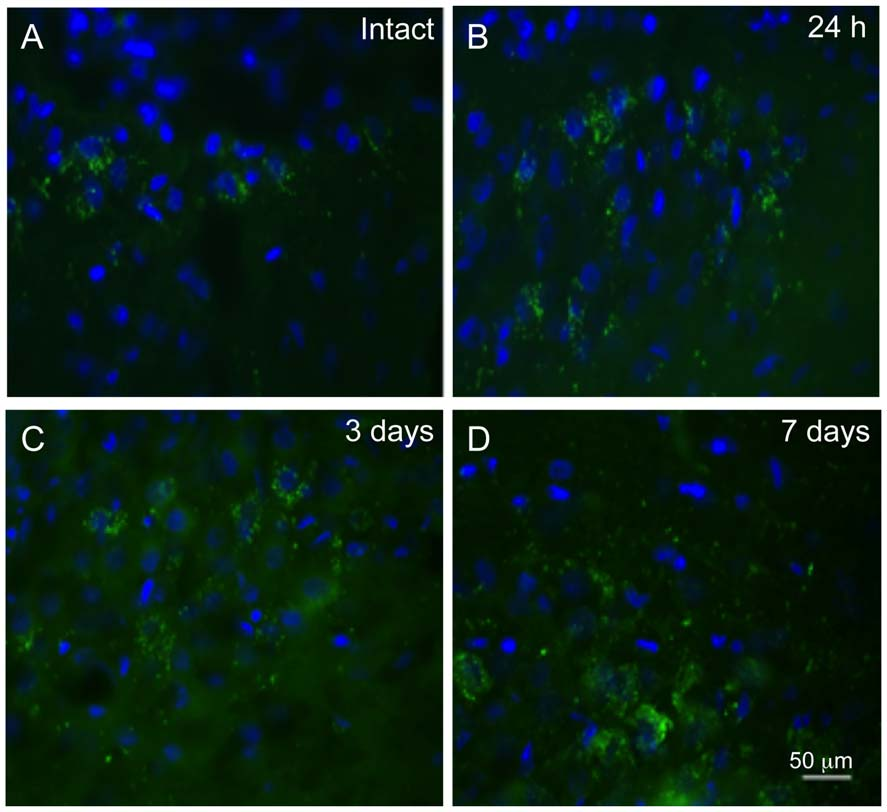
Augurin immunoreactivity in the supraoptic nucleus (SON) of the hypothalamus does not decrease following penetrating CNS injury. To demonstrate specificity of injury response in the CPe as opposed to other regions of the brain that have high levels of Ecrg4 gene and augurin protein expression [21], we examined the SON of the hypothalamus in injured rats at 1 (Panel B), 3 (Panel C) and 7 (Panel D) d.p.i. and compared staining in intact brain (Panel A). While there may have been a slight incrase at 3 and 7 d.p.i., the precipitous decline of expression that occurred in the CPe following injury was not observed in SON. Images representative of n = 3 rats.

To examine whether augurin levels change in a pattern similar to the CPe following cortical lesion, we looked for immunoreactivity in another area of the brain with high levels of augurin and Ecrg4 expression, the SON of the hypothalamus [21]. We found no decrease in expression at 1 and 3 d.p.i. in injured rats (Figure 4B and C) compared to control Figure 4A). This regional comparison indicates the decreased levels of augurin at 1 and 3 d.p.i. are not a global response throughout the brain, and further comparisons of anatomical regions will determine if this particular pattern is unique to the CPe in response to CNS injury.

## Discussion

The data presented here establish that CP-derived Ecrg4 is an injury response gene. First, CNS injury precipitates a rapid loss of Ecrg4 gene expression within 24 hours of injury and it remained sustained for 7 days, at which time the injury has begun to resolve [22]. The immediate response however, was presumably an initial bulk release of augurin from the CPe cell surface followed by more sustained secretion and release of the intracellular stores of augurin. The function of this bulk release in the earliest phase of injury is not known but it was followed by a depletion of immunoreactivity during the mid-phase of injury that was exacerbated by a down regulation of Ecrg4 gene expression that is sustained throughout the proliferative phase of CNS injury through to resolution at day 7.

The Ecrg4 gene is a candidate tumor suppressor gene that has gained significant attention because of the inverse association of gene expression with the growth and progression of epithelial cancers [23], [24]. Down regulation is mediated by its epigenetic silencing via DNA methylation of the Ecrg4 promoter [25], [26], [27]. Recently, Ecrg4 gene expression has also been inversely correlated with cancer progression and invasiveness [27], anti-inflammatory gene regulation through NF-kB [25], cell proliferation in the SVZ [15] and directly correlated with increased senescence in the CNS [15] and endocrine regulation of peripheral hydro-mineral balance [21], [28]. Its open reading frame (ORF) encodes a 148 amino acid protein that is highly conserved amongst species and has secondary structure motifs that characterize neuropeptides [13]. However, database mining reveals that Ecrg4 is not part of a larger family of related genes. This could indicate tight evolutionary control amongst Ecrg4 orthologs and a unique biological function.

The decrease in augurin protein (Figure 2) and Ecrg4 gene expression (Figure 3) occurs at times when cells proliferate in the normal CNS injury response [29]. The time course of decreased augurin correlates with the acute and sub-acute response times to CNS injury [1] as both gene and protein expression are decreased by 1 to 3 days post injury but return to uninjured levels by day 7. This is consistent with our previous demonstration [14] that Ecrg4 gene expression is high in the normal CP and that Ecrg4 overexpression compromises neuroprogenitor cell growth and differentiation. The findings presented here suggest that augurin may be like a “sentinel” quiescence factor whose constitutive presence is inhibitory, but whose absence becomes a dysinhibitory signal that enables the proliferative response to injury.

The fact that Ecrg4 gene expression is epigenetically regulated by DNA methylation raises the interesting possibility that the constitutive levels of augurin in CP might be controlled epigenetically as well. If so, DNA methylation of Ecrg4 would control the amounts of basal augurin that are constitutively present and available for release during the acute stage of injury. The significance of this hypothesis is not clear but it might signify that DNA methylation could also gauge the return of gene expression during the resolution phase of injury. Similar epigenetic modifications in DNA methylation are now well described after CNS ischemia and injury [30]. If reversal of such changes influences recovery of augurin expression levels, then in view of the effects of Ecrg4 gene knock down and over expression [30], neuroprogenitor responsiveness to injury could be significantly affected.

It is particularly noteworthy that, while augurin is similar to other peptide hormones in that it is constitutively produced by the CPe for secretion into CSF [22], it is also distinct from peptide hormones in that cell surface tethering (Figure 1C) is reminiscent of paracrine factors like epidermal and fibroblast growth factors, notch, jagged and ephrins [31], [32], [33], [34]. We [14] and others [15], have previously described a release of augurin into conditioned medium after transduction with Ecrg4 transgene, and we have now shown detection of a 14 kDa form in rat CSF, the first evidence of secreted augurin in vivo. Our findings of two different forms of augurin produced by CPe, 6 kDa (in vitro) and 14 kDa (in vivo), raise the possibility of different mechanisms of release. The fact that two different immunoreactive forms were detected could be a consequence of cell biology altered by the cell culturing process, and it is possible that in response to injury the 6 kDa is shed into CSF, while the 14 kDa form in Figure 1B is constitutively released. The isoform detected could be dependent on cell type and conditional: we have detected both isoforms in the conditioned medium of transduced cultured prostate epithelial cells (XD, manuscript in review) and the 6 kDa form in an ex vivo model of inflammation (AK, manuscript in preparation). Figure 1C supports the hypothesis that a tethered 14 kDa form is shed by proteolysis into media. The form in CSF however (Figure 1B) could be released by (1) removal of the membrane anchor, (2) constitutive release from the ER/golgi pathway or (3) by another cell type of origin.

**Figure 5:**
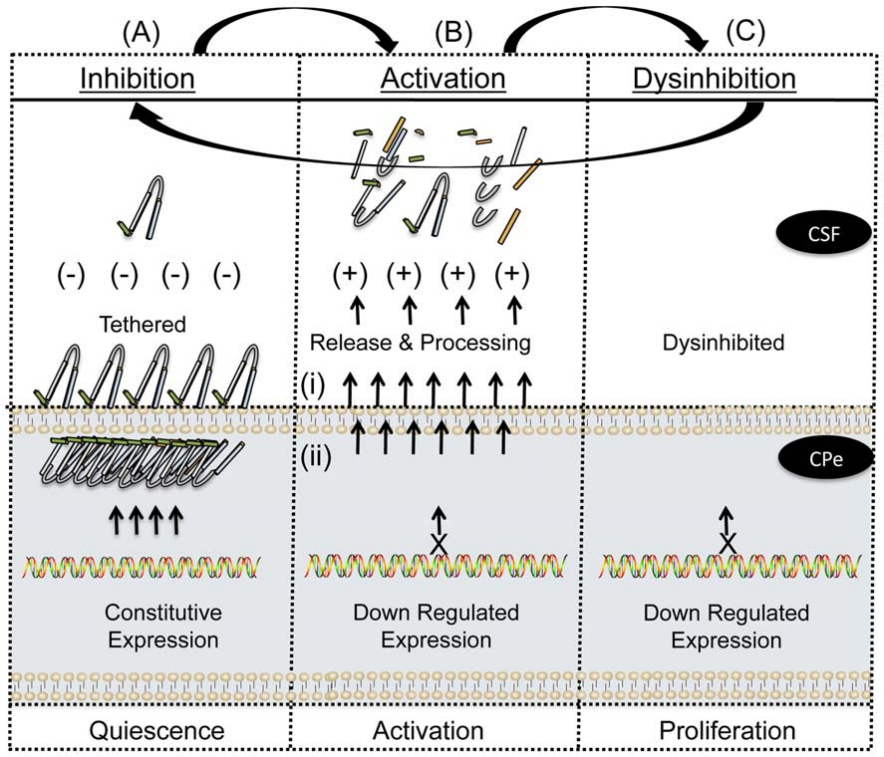
A biphasic model for augurin activity and CNS dysinhibition. Normal choroid plexus epithelia express Ecrg4 and contain significant 14 kDa augurin protein that is in an inhibitory conformation. The unprocessed peptide is contained in 3 compartments (1) intracellular vesicles (i.e. punctate intracellular staining in Figure 1) (2) near the ventricular cell surface (i.e. polarized apical staining of cells in Figure 1 and (3) tethered at the cell surface (i.e. Figure 1B). Upon injury, the sudden release and processing at the cell surface is followed by release from intracellular stores as a “panic signal” to indicate systemic injury. The initial pro-inflammatory response then shuts down Ecrg4 gene expression for at least 1 to 3 days post-injury. Therefore, during the initial phase of injury, its normal, constitutively inhibitory functions are absent and so repair cells proliferate. As gene expression returns, quiescence is restored and homeostasis re-established.

The loss of augurin immunoreactivity in CPe tissue following injury suggests that there are three pools of augurin: (1) peptide released into biological fluids such as CSF, (2) protein retained at the cell surface and (3) peptides stored in intracellular vesicles. The immediate response to injury is presumably processing at the cell surface and followed rapidly by augurin release from intracellular storage vesicles. Gene expression levels following injury (Figure 3) mirror protein levels (Figure 2) suggesting that the augurin released shortly after injury is not replenished. The observation that a smaller, processed form of augurin is found in conditioned media (Figure 1B, panel 3) suggests that proteolysis and processing occur at the time of cell surface shedding. The biological function, if any, of these fragments are not known but they may be distinct from that of intact augurin tethered at the cell surface (Figure 5).

The results presented here are compatible with a dual function model of augurin activity (Figure 5). Augurin normally acts as a sentinel inhibitory factor that is constitutively present on the epithelial cell surface but at early times after injury, augurin fragments generated from the cell surface may be a permissive signal for injury activation. The local decrease in augurin concentration would dysinhibit proliferation and migration of neuroprogenitor cells in the SVZ. Indeed, augurin overexpression in CP and ependyma decreases BrdU uptake and nestin immunoreactivity here, indicating that CPe-derived augurin regulates the proliferation and lifespan of neuroprogenitors [3], [35], [36]. Its dysinhibition could help divert progenitor migration to the injury site from the rostral migratory stream [3], [35], [36]. If so, the epigenetic regulation of Ecrg4 gene expression in the CP could help guage the delicate balance between neuroprogenitor cell mobilization in the SVZ for repair and repression of grown inhibition. . Further kinetic analyses of augurin distribution and Ecrg4 expression during the acute and chronic phases of recovery from cortical lesions will address this possibility.

## Materials and Methods

### Animals

All studies using rats were conducted with prior approval of, and in accordance with, either the Institutional Animal Care and Use Committee at Brown University (Providence, RI, approval numbers 0062-07 and 0073-10) or the Home Office at Birmingham University (Edgbaston, UK, approval number 3012720-19b2). All survival surgeries were conducted under aseptic conditions. Tissue was harvested from adult male Sprague Dawley rats maintained in standard light/dark conditions with *ad libitum* access to food and water. For brain tissue harvesting, rats were killed by CO_2_ inhalation and immediately perfused with 4% paraformaldehyde (PFA) in phosphate-buffered saline (PBS). Brains were removed and further fixed overnight in PBS containing 20% sucrose and 4% PFA at 4°C. The, brains were incubated in PBS containing 30% sucrose overnight and frozen in OCT compound on dry ice and stored at −80°C until further processing.

### Antibodies

#### Immunofluorescence

A polyclonal IgY antibody was raised in chickens against recombinant human ECRG4(71–148) and antigen affinity purified by commercial contract with GenWay Biotech, Inc., (San Diego, CA). Purified pre-immune IgY from the same animal was used as a negative control in immunostaining of rat brain tissue and goat anti-chicken-AlexaFluor® 594 (Life Technologies, Carlsbad, CA) was used for detection. Western blotting: Affinity purified rabbit anti- human ECRG4 primary antibody (Sigma-Aldrich, St. Louis, MO) and Goat anti-rabbit–Horseradish Peroxidase (HRP) conjugated secondary antibody (JacksonImmuno Labs, West Grove, PA) were used to detect protein in Western blotting analysis.

### Viral vectors

An adenovirus vector containing a transgene for the human Ecrg4 ORF (AD_ECRG4_) was prepared as described previously. An adenovirus containing the transgene for green fluorescent protein (AD_GFP_, Vector BioLabs, Philadelphia, PA) was used as a control.

### Cell Culture

A primary human CP epithelial cell line was purchased from ScienCell Research Laboratories (Carlsbad, CA) and cultured in Epithelial Cell Medium according to manufacturer's instructions. For adenovirus infection, confluent cells were counted and transduced with a multiplicity of infection (MOI) of 20.

### Penetrating CNS Lesion Model

Rats were anesthetized with 5% isoflurane in oxygen (1.7 L/min) and given 0.3 mg/kg buprenorphine subcutaneously for analgesia prior to CNS injury. Anesthesia was assessed by paw pinch reflex. The skull was first exposed with a sagital incision along the midline of the head and after creating a burr hole through the dura with a dental drill, a lesion was made 3 mm right of bregma, 7.5 mm rostral of the ear-bar plane and 3 mm deep using an ophthalmic knife (Unitome Knife, BD Waltham, MA). The injury inflicted transected the corpus callosum and the knife wound entered the striatum. We have used this injury model extensively in other studies [37], [38], [39], [40]. Intact rat brains from un-operated animals were used as controls.

### Collection of rat CSF

CSF samples from adult Spragu-Dawley rats were collected as described previously [41]. Briefly, rats were deeply anaesthetized in a stereotaxic frame with the head raised above the body. An incision was made from the top of the head to the base of the neck. The muscle was retracted from around the base of the skull. Using a Hamilton syringe, CSF was withdrawn from the cisterna magna and immediately frozen on dry ice. Rats were then killed by terminal anesthesia.

### Cell Surface Protein Fractionation and Immunoblotting

Primary human CPe cells were infected with AD_ECRG4_ or AD_GFP_ a MOI of 20 to overexpress either Ecrg4 or GFP. Cell surface proteins were biotinylated and pulled down using the Cell Surface Protein Isolation Kit according to manufacturer's instructions (Pierce, Rockford, IL). Precipitated proteins were eluted from Neutravidin beads by incubating in 2% lithium dodecyl sulfate sample buffer under reducing conditions for one hour at 25°C. Immunoreactive protein was detected by Western blotting. Briefly, proteins were size fractionated on a 4–12% Bis-Tris gel for SDS-PAGE. Proteins were transferred to 0.2 µm polyvinylidene fluoride membrane, which was blocked with 5% bovine serum albumin (BSA) solution for one hour at 25°C. Rabbit anti-human augurin (Sigma-Aldrich, St. Louis, MO) was diluted in 1% BSA at a concentration of 0.5 µg/ml and incubated with the membrane overnight at 4°C. Following washes with PBS containing 0.05% Tween-20, the membranes were incubated with 0.1 µg/ml goat anti-rabbit-HRP secondary antibody diluted in 1% BSA for one hour at 25°C, rinsed and then incubated with Super Signal West Pico Chemiluminescent Substrate (Pierce, Rockford, IL). Bioluminescence signal was detected by imaging with an IVIS® Lumina imaging system (Caliper Life Sciences, Hopkinton, MA).

### Immunostaining of Rat Brain Tissue

Frozen rat brains with and without lesions were cut into 15 µµm sections for immunohistochemistry. Briefly, non-specific protein binding was blocked in PBS containing 0.3% Tween-20, 2% BSA and 15% normal goat serum. Samples were rinsed and incubated overnight at 4°C with 0.5 µg/ml chicken anti-augurin antibody. After washing, sections were incubated with either 1 µg/ml goat anti-chicken Alexa Fluor® 594 (Figure 1) or Alexa Fluor® 488 (Figure 2) secondary antibody for 45 min at room temperature. Sections were rinsed and mounted with Vectashield containing DAPI (Vector Labs, Burlingame, CA).

### In Situ Hybridization

Sections from control and injured rats were processed and sectioned as for immunohistochemistry. Digoxigenin labeled antisense RNA probe to rat Ecrg4 cDNA was synthesized with the DIG RNA Labeling Kit (Roche, Indianapolis, IN) and hybridized to tissue as described previously [14].
